# Identification of Dwarfing Candidate Genes in *Brassica napus* L. LSW2018 through BSA–Seq and Genetic Mapping

**DOI:** 10.3390/plants13162298

**Published:** 2024-08-18

**Authors:** Sha Huang, Fang Wang, Yang Li, Zhuanzhuan Wang, Ruimao Zhang, Jijun Li, Chao Li

**Affiliations:** 1 Guizhou Oil Crops Research Institute, Guizhou Academy of Agricultural Sciences, Guiyang 550006, China; 2 Ministry of Agriculture and Rural Affairs, Key Laboratory of Crop Genetic Resources and Germplasm Innovation in Karst Region, Guiyang 550006, China; 3 The Key Laboratory of Plant Resource Conservation and Germplasm Innovation in Mountainous Region, Ministry of Education, Guizhou University, Guiyang 550025, China

**Keywords:** LSW2018, BSA–seq, dwarf, candidate gene

## Abstract

Plant height, as a crucial component of plant architecture, exerts a significant influence on rapeseed (*Brassica napus* L.) lodging resistance, photosynthetic efficiency, yield, and mechanized harvest level. A previous study identified dwarf rapeseed LSW2018. In this study, LSW2018 (dwarf parent (PD)) was crossed with 389 (high parent (PH)) to establish the F_2_ population, and 30 extremely dwarf (bulk–D) and high (bulk–H) plants in the F_2_ population were respectively selected to construct two bulked DNA pools. Whole–genome sequencing and variation analysis (BSA–seq) were performed on these four DNA pools (PD, PH, bulk–D, and bulk–H). The BSA–seq results revealed that the genomic region responsible for the dwarf trait spanned from 19.30 to 22.19 Mb on chromosome A03, with a length of 2.89 Mb. After fine mapping with simple sequence repeat (SSR) markers, the gene was narrowed to a 0.71 Mb interval. Within this region, a total of 113 genes were identified, 42 of which contained large–effect variants. According to reference genome annotation and qRT–PCR analysis, there are 17 differentially expressed genes in this region between high and dwarf individuals. This study preliminarily reveals the genetic basis of LSW2018 dwarfing and provides a theoretical foundation for the molecular marker–assisted breeding of dwarf rapeseed.

## 1. Introduction

Rapeseed is one of the main oil crops in the world, and yield improvement is an important goal of rapeseed breeding. The utilization of heterosis is an important measure to improve yield in rapeseed and other crops [1,2,3,4]. Studies have demonstrated that heterosis also increases plant height [5,6,7,8], and plant height has a significant positive correlation with the lodging index [9]. Therefore, the overgrowth of plants may also lead to increased lodging risk in rapeseed. Lodging not only affects rapeseed yield and seed quality but also increases the challenge of mechanized harvesting [10,11,12]. The “Green Revolution” in the 1960s led to a significant increase in the yields of major staple grain crops (wheat, corn, and rice) through the widespread adoption of semi–dwarf plant varieties [13,14]. Considering the significant achievements of the “Green Revolution”, the excavation and utilization of dwarf and semi–dwarf plant resources have attracted the attention of breeders.

The key to rapeseed semi–dwarf breeding lies in the acquisition of germplasm resources and the identification and utilization of dwarfing genes. In recent years, a series of dwarf and semi–dwarf rapeseed germplasm resources have been created by natural mutation, interspecific hybridization, and physical mutagenesis, such as YA2016–12 [15], ds–3 [16], ds–4 [17], sca [18], G7 [19], and H2M–1 [20]. Studying the genetic mechanism of dwarf material and identifying its dwarfing genes can contribute to the reduction of plant height and the improvement of lodging resistance. At present, considerable progress has been made in the study of the rapeseed dwarfing mechanism. Some genes related to dwarfing have been mapped and identified, including *BnaDwf.C9* [21], *Bzh* [22,23], *DS–1* [24], *DS–3* [16], *DS–4* [17], *G7* [19], *BND2* [25], *BnaC04.BIL1* [26], *BnaA03.BP* [27], etc. It has been reported that the underlying mechanisms of these genes are primarily associated with plant hormone signaling transduction and biosynthesis. Although there have been numerous achievements in the exploration of dwarf breeding and the mechanism of dwarfism in rapeseed, it is still necessary to explore new dwarfing materials and identify new dwarfing genes. This will help to increase the genetic diversity of dwarfing materials and provide an effective strategy for cultivating “ideotype” rapeseed and increasing rapeseed yield.

With the advancement of next–generation sequencing technology in recent years, whole–genome sequencing and bulked segregant analysis (BSA–seq) have been widely used in the gene mapping and molecular marker development of crops, such as maize (*Zea mays* L.) [28,29], watermelon (*Citrullus lanatus*) [30], squash (*Cucurbita pepo*) [31], rice (*Oryza sativa*) [32], cotton (*Gossypium hirsutum* L.) [33], rapeseed (*Brassica napus* L.) [25], peanut (*Arachis*) [34], and *Brassica rapa* ssp. *chinensis* Makino [35]. For instance, using BSA–seq, *AhRt*, a candidate gene controlling peanut red shell, was identified on chromosome 22 and further narrowed the mapping interval to the 530 kb region by linkage mapping with 220 recombinant lines [34]. Through BSA–seq analysis of 638 F_2:3_ individuals, a new height–related quantitative trait loci (QTL) (*qPH9*) was identified, which was mapped to a 2.02 Mb region of chromosome 9. Using 13 Kompetitive allele–specific PCR (KASP) markers combined with traditional linkage analysis facilitated the localization of *qPH9* to a 126 kb region [32]. In order to map the dwarf gene *BND2*, BSA–seq was performed on 25 extremely dwarf and 23 extremely high individuals selected from the F_2:3_ population, and the candidate interval was rapidly identified to locate in the range of 13.77–18.08 Mb on chromosome A08 with a length of 4.31 Mb [25]. BSA–seq improves the accuracy and efficiency of locating major QTLs and mining target genes when combined with traditional gene mapping methods.

In a previous study, we identified a dwarf mutant in rapeseed, LSW2018. The average plant height of LSW2018 was 136.24 cm after transplanting. In addition, LSW2018 has a dark leaf color, thick wrinkled leaves, short petiole, short internode, and low branch position, which we call the “dwarf” phenotype of LSW2018 [36]. The dwarf trait of LSW2018 is controlled by a major dominant gene, without cytoplasmic effects [37]. In order to identify the chromosome position of the dwarf gene, LSW2018 and 389 were hybridized to construct an F_2_ segregation population for genetic research in this study. Then, BSA–seq, combined with QTL mapping, was used to identify the dwarf gene. Based on the above research, we aimed to investigate the genetic basis of the dwarf trait in LSW2018, thereby providing a theoretical foundation for the utilization of the dwarf gene and breeding of dwarf rapeseed.

## 2. Results

### 2.1. Sequencing Data Statistics and Analysis

To identify the genes controlling the dwarf trait of LSW2018, we constructed a heterozygous F_2_ population using dwarf parent LSW2018 and high parent 389. Whole–genome re–sequencing was performed on two parents, LSW2018 (PD) and 389 (PH), as well as bulk–D and bulk–H. According to the BSA–seq results, sequencing data quality control analysis was conducted, and a total of 122.67 GB of data were obtained from four samples after filtering. In the clean data, the number of clean reads was 421,417,286, with the Q30 values ranging from 90.24% to 94.05%, Q20 values ranging from 96.21% to 98.16%, and the GC content ranging from 37.49% to 38.27% (Table 1).

### 2.2. Statistics of Sequencing Alignment Results

The comparison analysis using BWA software (version 0.7.15–r1140) showed that the mean clean read alignment rate of the four samples with the ZS11 reference genome was 96.26%, indicating that the library was free of contamination and constructed normally (Table 2). After the reads were mapped onto the reference genome, the base coverage rate and coverage depth on the reference genome were statistically analyzed. The results show that the genome coverage rate of each sample was above 85%, indicating great integrity of the variation detection. The sequencing depth ranged from 19.5x to 23.29x, indicating high accuracy of the variation detection (Table 3). In summary, the amount of data from the sequenced samples was sufficient, with good quality, and the sequencing depth coverage met the requirements for the subsequent analysis.

### 2.3. Detection of Polymorphic Loci

**Figure 1:**
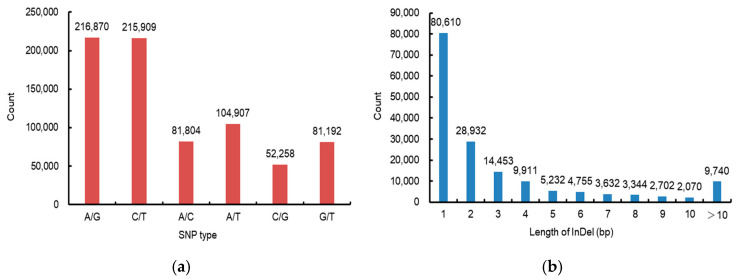
Variation type statistics: (**a**) SNP type statistics; (**b**) InDel length statistics.

After SNP and InDel detection, a total of 918,321 variations were screened from the four DNA pools. Among these variation sites, 752,940 were SNPs and 165,381 were InDels. Among the SNPs, 432,779 were transition types (A/G and C/T), and 320,161 were transposable types (A/C, A/T, C/G, and G/T). The transition transposable ratio (Ts/Tv) was 1.35 (Figure 1a). Among the InDels, 80,610 are 1 bp in length, 28,932 InDels are 2 bp in length, and a total of 55,839 are not less than 3 bp in length (Figure 1b).

**Figure 2:**
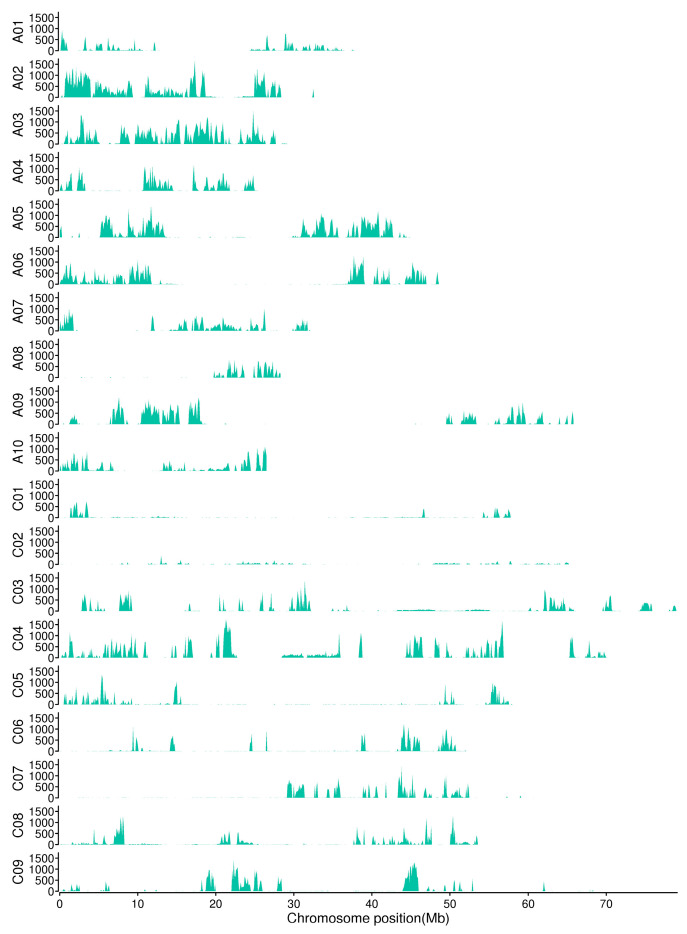
Distribution of SNPs/InDels on chromosomes (statistics in 200 kb windows).

In analyzing the number and density of variations in each chromosome, it was found that the average density of SNP on the chromosomes was 783.64/Mb, and the average density of InDels on the chromosomes was 172.12/Mb (Table 4). A sliding window of 200 kb was used to calculate the distribution of available SNP/InDel variations on the chromosomes; the results show that these variations were widely distributed on 19 chromosomes (Figure 2).

### 2.4. Determination of Candidate Region

**Figure 3:**
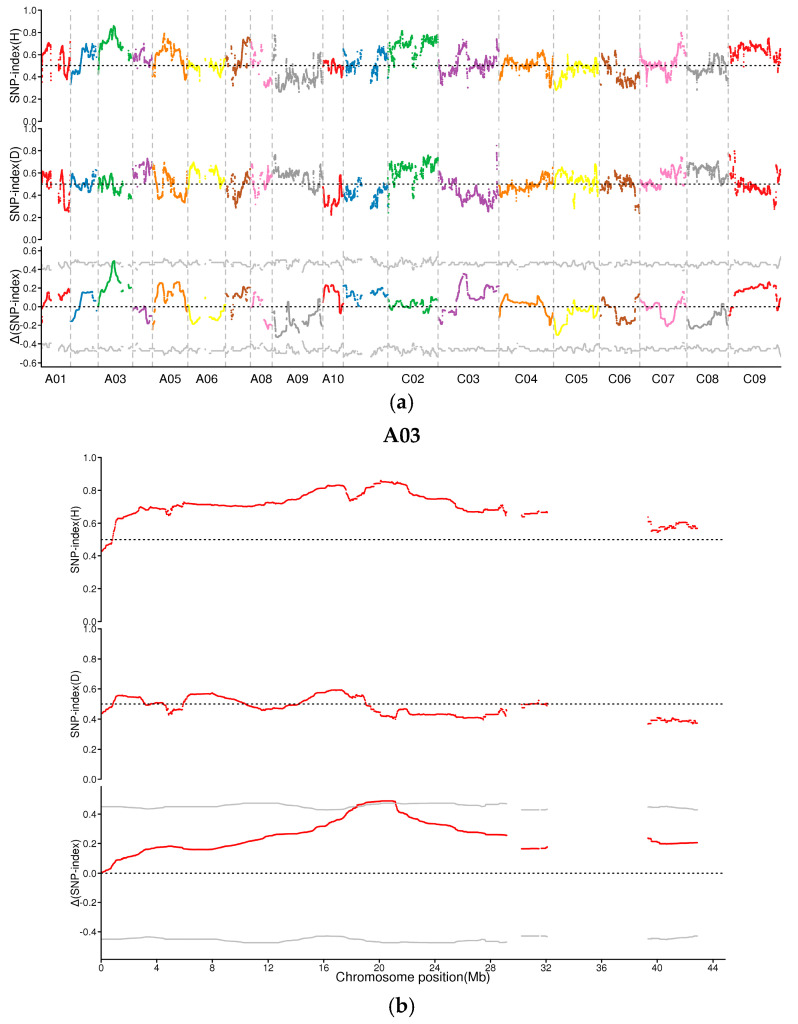
Preliminary BSA sequencing results of dwarf mutant LSW2018. (**a**) Distribution of Δ(SNP–ndex) values on each chromosome; (**b**) candidate interval of dwarf gene LSW2018. The horizontal gray line is the threshold (the corresponding *p*–value is 0.01).

The SNP indexes of the dwarf and high materials were obtained, and the Δ(SNP–index) values were calculated based on the two kinds of materials (Figure 3a). A sliding window of 2 Mb was used to calculate the Δ(SNP–index) value of each variation and analyze its distribution on the genome through QTLseqr (R package). The results show that the region of the dwarf gene was located at 19.30–22.19 Mb on chromosome A03, with a length of 2.89 Mb (Figure 3b). There were a total of 8119 variation loci in this candidate interval, including 6311 SNPs and 108 InDels. The total number of coding genes in this interval was 473, and 124 of these genes had large–effect variants (Table 5).

### 2.5. Fine Mapping Using SSR Markers

**Figure 4:**
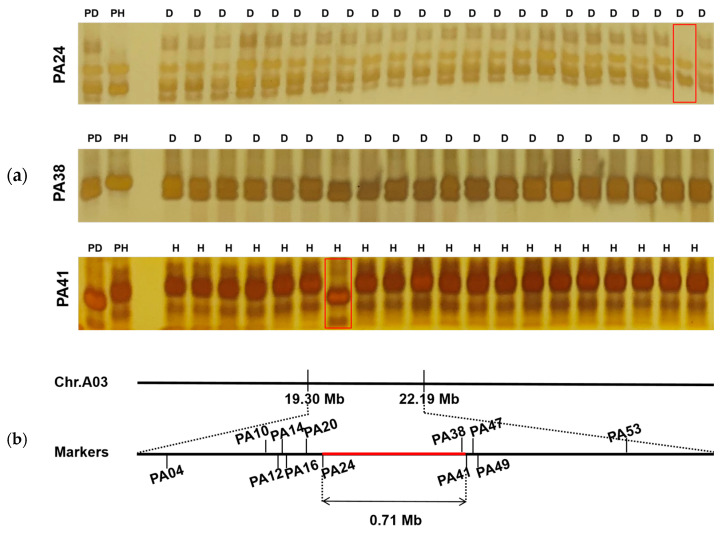
Map–based cloning of dwarf traits with SSR markers. (**a**) Amplification results of partial SSR primer polymorphisms in high and dwarf individuals. Note: PD: dwarf parent (LSW2018), PH: high parent (389), D: dwarf individuals, H: high individuals, the individuals in the red box are recombinants; (**b**) linkage map of the SSR markers.

In order to verify the accuracy of the position and narrow the candidate region, we developed SSR markers within the 2.89 Mb candidate region for further fine mapping. Based on the BSA–seq, we first screened the polymorphic SSR loci using sequences of two parents (LSW2018 and 389), and 30 SSR markers were identified and designed using Primer5 (Table S1). Seventeen SSR markers showed polymorphism between the two parents. We re–identified the polymorphisms of 17 SSR markers in a tiny F_2_ population (30 individuals), and 14 SSR markers were polymorphic in this population. Finally, these 14 markers were verified in the F_2_ population with 548 individuals. Two markers had poor polymorphism results in the population with 548 individuals, and the remaining 12 SSR markers (Table S2) were used for subsequent genetic analysis. The genotypic and phenotypic analysis of the 548 individuals narrowed the dwarf–associated region to the segment delimited by markers PA24 and PA41 (Figure 4a). According to the physical location of the SSR markers on chromosome A03, the dwarf gene was determined to be located at the 20.23–20.94 Mb region, with a range of 0.71 Mb (Figure 4b).

### 2.6. Functional Annotation of Candidate Genes

According to the rapeseed genome database, a total of 113 genes were found in the 20.23–20.94 Mb region on chromosome A03. Combined with the BSA–seq results, 42 genes were found to have non–synonymous mutations or frameshift mutations in the exon region with large–effect variants (LEVs) (Table 6, Table S3).

**Figure 5:**
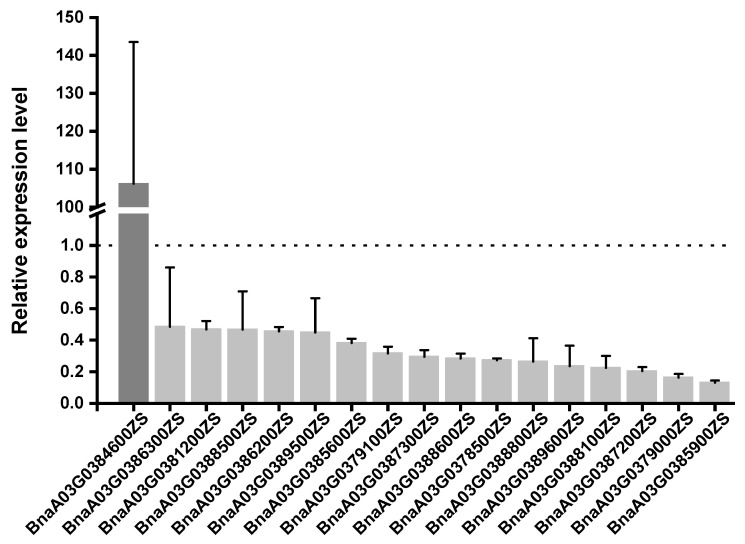
The expression levels of 17 different genes in dwarf and high individuals. Note: The Y–axes indicate the relative expression quantity. The error bars indicate the SD for the data obtained from three biological replicates.

To investigate the differential expression levels of the 42 annotated genes and identify the candidate gene, the transcript levels of these genes were examined via qRT–PCR in dwarf and high individuals from the separated population. The expression levels of 11 genes were barely detected in both the dwarf and high individuals, 14 genes were without differences (fold–change < 2), and 17 genes were differentially expressed between the two kinds of individuals (Table S4). Among the 17 differentially expressed genes, BnaA03G0384600ZS had higher transcript levels in the dwarf individuals than the high ones (by about 106–fold), and the other 16 genes transcript levels were lower in the dwarf individuals than the high ones (fold–change > 2) (Figure 5).

## 3. Discussion

In this study, we identified a dwarf rapeseed with potential for application in production. Research on crop dwarfing has shown that dwarf plants can reduce the incidence of lodging, increase the grain number per spike, and improve the harvest index, thereby resulting in amplified crop yield and heightened grain quality [13,32,38]. However, the excessive dwarfing of plants results in the overlapping of canopy leaves, a reduction in biomass and yield, and decreased disease resistance [39]. Thus, extremely dwarfed plants are not conducive to production and utilization. Therefore, rapeseed breeders commonly believe that the semi–dwarf (1.2–1.5 m) and compact type may be the “ideotype”, which can ensure sufficient biomass, lodging resistance, and harvest efficiency [27,40]. The dwarf material LSW2018 developed in this study has an average plant height of 136.24 cm in the field, belongs to semi–dwarf rapeseed, and is resistant to dense planting and suitable for mechanized production. Therefore, LSW2018 is an excellent rapeseed germplasm resource with potential production application value.

BSA–seq, combined with traditional gene mapping methods for genetic analyses, is a simple and rapid approach that can effectively narrow the major QTL interval. Compared to BSA–seq, traditional QTL analysis is a time–consuming and laborious process. The genome–wide development of molecular markers is necessary, and multiple segregating populations need to be constructed for candidate region screening and gene fine mapping [41]. In genetic studies, BSA only requires the selection of extreme phenotypic individuals or representative individuals in the population to construct mixing pools for QTL identification and gene mapping [42]. In different crops, BSA–seq technology combined with traditional gene mapping methods has been widely used to identify genes controlling the accumulation of epicuticular waxes [28], sex determination [30], disease resistance [31], plant height [32], branch [33], and dwarfing [25,29]. In our study, through BSA–seq and traditional mapping methods, the genes related to dwarf traits in LSW2018 were accurately delimited to chromosome A03 within two years. Due to the BSA–seq method, only an F_2_ population containing 548 individuals was used in the gene mapping, and the genetic region was narrowed to a 0.71 Mb region. These results indicate that BSA–seq is an effective and efficient method of genetic analysis.

A total of 42 genes were identified by QTL mapping and BSA–seq in the candidate region, of which 17 genes were differentially expressed in this region between high and dwarf individuals. Among them, BnaA03G0384600ZS had a higher expression level in the LSW2018 than HSW2018 (about 106–fold), and is predicted to encode long–chain acyl–CoA synthetase 8 (*LACS8*). In *Arabidopsis*, both lacs4 and lacs8 have an impact on cuticular lipid metabolism. After the *lacs4lacs8* double mutation, the overall size of the plant was reduced compared to the wild type, and the leaves exhibited characteristics such as being short, rounded, curled, and disordered [36,43]. In this study, the LSW2018 mutant also displayed a similar phenotype. Subsequently, we conducted chlorophyll leaching analysis and observed that the LSW2018 mutant exhibited higher cuticle permeability than HSW2018 (Figure S1). Based on these findings, we hypothesized that the loss of function of BnaA03G0384600ZS may result from defects in cutin or wax deposition, consequently leading to corresponding phenotypic changes. However, this study found that the expression level of BnaA03G0384600ZS was increased in dwarf individuals. According to the report, after point mutations or deletion derivatives, one function of a protein will be inactivated, but the ability to interact with other large molecules will still be preserved, which might cause mutant phenotype by competition [44,45]. Therefore, we hypothesized that BnaA03G0384600ZS may have similar functions after mutation, and the enhanced expression makes LSW2018 exhibit a distinct mutant phenotype. *Brassica napus* is an allotetraploid crop with a complex genome, which leads to potentially complex interactions between genes [46]. These results cannot fully confirm that functional alterations in this gene are responsible for the dwarfing phenotype of LSW2018. We will further explore the role and mechanism of *LACS8* in the formation of the LSW2018 dwarfing phenotype in subsequent studies.

Numerous studies have demonstrated that plant hormones, including auxin [47], gibberellin [48,49], brassinolide [50], and strigolactone [51] play a key role in regulating plant height. Based on gene function annotation, we found 16 genes have lower expression levels in dwarf individuals, including 2 hormone–related genes, BnaA03G0386300ZS and BnaA03G0388800ZS. BnaA03G0386300ZS is predicted to encode cytochrome b561, a member of the auxin–responsive family protein involved in auxin signaling. Mutation of the cytochrome b561–encoding gene *OsCYBDOMG1* exhibited a dwarfing phenotype in rice [52]. BnaA03G0388800ZS is predicted to encode cycloartenol synthase, which plays a crucial role in the biosynthesis of brassinosteroids (BRs). BRs are a class of plant steroid hormones that play key roles in regulating plant growth and development. When BR synthesis and signal transduction pathways are blocked, plants will exhibit a dwarfing phenotype [53]. The BR content was reduced in the pea mutant lk after mutation of the BR synthesis–related gene *DET2*, resulting in plant dwarfing [54]. Mutation of the BR signaling pathway–related genes *RLP44* and *RBI1* also led to a dwarfing phenomenon in *Arabidopsis thaliana* plants [55,56]. Other genes may also be involved in regulating the dwarfing of LSW2018, but further experiments are necessary to confirm the functions of these genes.

## 4. Materials and Methods

### 4.1. Population Construction and Traits Investigation

LSW2018, 389, and two generations (F_1_ and F_2_) of their hybrid constructs were planted at the Guizhou Academy of Agricultural Sciences, with row spacing of 0.4 m and plant spacing of 0.33 m. The field planting and management of the experimental materials followed the farmers’ habits. The F_2_ segregating population was established, comprising approximately 548 individual plants, which exhibited a phenotypic ratio of 3:1 between the dwarf and high groups.

### 4.2. Whole–Genome Re–Sequencing

Bulked segregant analysis (BSA) [57] was preliminarily used for the dwarf gene mapping. At the seedling stage, 10 plants of LSW2018 (dwarf parent, PD) and 389 (high parent, PH) were selected for constructing two parent DNA pools, and 30 extremely dwarf (bulk–D) and high (bulk–H) plants in the F_2_ population were selected for constructing two bulked DNA pools. The tender leaves of the plants were sampled in 1.5 mL centrifuge tubes stored in dry ice, and then sent to the Genoseq Corporation (Wuhan, China) for database construction and sequencing. The specific methods and procedures are as follows:

The total DNA was extracted using the CTAB method [58], and RNase was used to remove the RNA from the extracted DNA samples. The purity and integrity of the DNA samples were assessed using agarose gel electrophoresis, ensuring that the genomic DNA remained intact and without RNA contamination. The DNA concentration was detected using the NanoDrop 2000c (Thermo Scientific, Waltham, MA, USA), and the final mass concentration was adjusted to 40 ng/μL. The library was constructed according to a standard procedure, and the four DNA pools (PD, PH, bulk–D, and bulk–H) were extracted for whole–genome re–sequencing.

The bioruptorUCD–200 was used to process the qualified DNA samples through quality testing to obtain fragments with lengths between 200 bp and 500 bp. Then, the fragmented DNA was treated with terminal repair and A tail and then connected to the sequencing connector. Target fragments were selected according to their expected library size and purified to remove splice contamination. Sequencing libraries were constructed by PCR enrichment and purified using AMPure XP Beads. The quality of the library was tested, and the PCR products were subjected to paired–end 150 bp (PE150) sequencing on the Illumina HiSeq platform after passing the quality inspection. According to the above process, the four DNA pools (PD, PH, bulk–D, and bulk–H) were constructed and sequenced, respectively. The sequencing depth of the PD and PH pools was ~20x, and the sequencing depth of bulk–D and bulk–H pools was ~30x.

After sequencing, data quality control was performed based on the raw data. Cutadapt software (version 1.13) was used to remove the linker sequences in the reads, and trimmomatic software (version 0.36) was used to eliminate low–quality bases. The filtered clean reads were compared with the reference genome ZS11–v20200127 (download link: http://cbi.hzau.edu.cn/cgi-bin/rape/download_ext) (accessed on 23 February 2021) using BWA software.

### 4.3. Polymorphic Site Detection and Variant Annotation

All samples were tested for variation using the GenotypeGVCFs module of GATK (version 3.7) software, including single–nucleotide polymorphisms (SNPs) and insertion/deletion mutations (InDels). ANNOVAR software (version 2016Feb1) was used to annotate the variants and predict the effects of the variants on gene function. According to the method of Takagi et al. [41], the SNP frequency Δ(SNP–index) on each polymorphic locus was calculated using QTLseqr (R package). According to the distribution of the Δ(SNP–index) values throughout the genome, candidate gene intervals related to dwarfing traits were screened out.

### 4.4. Gene Localization

MISA software (version 2.1) was used to identify SSR loci in the reference genome and determine the location information of these loci. According to the results of the MISA software analysis, SSR markers were designed using Primer5 software. Combined with the preliminary localization results, the SSR markers were screened in the candidate region, and the primers were sent to Sangon Biotech (Shanghai, China) for synthesis. The specific primer sequences are shown in Table S1 in Supplementary Materials.

SSR markers with polymorphisms between parents were selected to detect polymorphisms in the 30 F_2_ individuals, and non–polymorphic markers were removed. Finally, 548 individuals were genotyped using the selected polymorphic markers. SSR amplification was performed in a final volume of 25 µL with a PCR mixture including 2 μL template DNA (50 ng·µL), 12.5 μL2 × Taq Master Mix for PAGE (Vazyme, Nanjing, China), 1 μL forward and reverse primers (10 μM), and 8.5 μL ddH_2_O. PCR was performed in a T100™ thermal cycler (Bio–Rad, Hercules, CA, USA) with the following program: initial denaturation at 95 °C for 3 min; 32 cycles of denaturation at 95 °C for 15 s; annealing at 56–62 °C (for different markers) for 15 s and extension at 72 °C for 60 s; and a final extension step at 72 °C for 5 min. PCR–amplified products were detected by 7% acrylamide gel electrophoresis and observed after rapid silver staining.

According to the phenotype and genotype results, the chromosome positions of the dwarf genes were determined with genetic analysis. Based on BSA–seq, genes with large–effect variants within the localization interval were identified according to the sequence polymorphisms between the dwarf and high materials. The BnIR rapeseed multi–omics database (https://yanglab.hzau.edu.cn/BnIR/) (accessed on 2 February 2024) and the Arabidopsis database (https://www.arabidopsis.org/) (accessed on 22 February 2024) were used to perform functional annotations on the genes with large–effect variants, and dwarf candidate genes were predicted based on the functional annotations.

### 4.5. Candidate Gene Prediction

Quantitative real–time PCR (qRT–PCR) was performed to identify the candidate genes. Total RNA samples were isolated from the leaves of dwarf and high individuals in the separated population using GeneJET (Thermo Fisher Scientific, Waltham, MA, USA), and first–strand cDNA was synthesized using a RevertAid™ Master Mix with DNase I (Thermo Fisher Scientific, Waltham, MA, USA). The reverse transcription products were used as templates for PCR, using the designed primers (Table S5) to examine the expression of the predicted genes. qRT–PCR was performed using the Genious 2X SYBR Green Fast qPCR Mix (ABclonal, Wuhan, China) and the Bio–Rad CF96 Real–Time system (Bio–Rad Laboratories, USA). Three biological replications were performed with three technical replicates for each sample. The comparative cycle threshold method was used to calculate the relative expression levels of the different genes. Rapeseed gene *BnACTIN7* was used to normalize the expression levels of the target genes, which were analyzed using the relative quantification method 2^−ΔΔCt^ [59].

## 5. Conclusions

Using BSA–seq and traditional genetic mapping methods, the genes related to dwarf traits in LSW2018 were successfully identified in a physical region of 0.71 Mb on chromosome A03. Through sequence large–effect variant analysis, qRT–PCR analysis, and gene functional annotation, 17 genes were identified as dwarfing candidate genes in LSW2018. These results will contribute to a better understanding of the genetic mechanism of rapeseed dwarfing and provide a theoretical foundation for the molecular marker–assisted breeding of dwarf rapeseed.

## Figures and Tables

**Table 1:** Statistical table of sequencing data.

Sample	Reads	Bases	GC (%)	Q20 (%)	Q30 (%)
PD	91,312,645	26,981,120,020	38.05	98.13	93.97
PH	98,731,000	29,170,655,657	38.27	98.16	94.05
Bulk–D	116,449,533	33,301,461,112	37.94	96.21	90.24
Bulk–H	114,924,108	33,216,959,201	37.49	96.80	91.46
Mean	105,354,322	30,667,548,998	37.94	97.33	92.43
Sum	421,417,286	122,670,195,990	–	–	–

Note: Sample: mixed pool sample number; Reads: number of clean PE reads measured; Bases: total number of clean bases measured; GC: GC content; Q20: percentage of bases with a mass fraction greater than 20; Q30: percentage of bases with a mass fraction greater than 30.

**Table 2:** Alignment rate of sequencing data.

Sample	Uniquely Mapped		Repeatedly Mapped		Unmapped	
	Count	Rate (%)	Count	Rate (%)	Count	Rate (%)
PD	54,925,179	60.15	32,399,172	35.48	3,988,294	4.37
PH	62,756,259	63.56	32,560,904	32.98	3,413,837	3.46
Bulk–D	69,785,881	59.93	42,508,895	36.50	4,154,757	3.57
Bulk–H	68,169,569	59.32	42,663,236	37.12	4,091,303	3.56
Mean	63,909,222	60.74	37,533,052	35.52	3,912,048	3.74

Note: Sample: sample number; Uniquely Mapped: reads mapped to a unique location on the genome; Repeatedly Mapped: reads mapped to multiple locations on the genome; Unmapped: reads that cannot be mapped to the genome.

**Table 3:** Genomic coverage and coverage depth statistics.

Sample	Coverage (%)	Mean Depth
PD	85.64	19.5
PH	86.83	22.07
Bulk–D	88.42	23.29
Bulk–H	88.81	22.85
Mean	87.42	21.93

Note: Sample: sample number; Coverage: percentage covering the entire genome; Mean Depth: average coverage depth.

**Table 4:** Statistical table of variation number and density.

Chr	Length	No. of SNPs	SNP Density	No. of InDels	InDel Density
A01	38,004,428	18,441	485.23	4560	119.99
A02	35,943,954	73,111	2034.03	17,176	477.86
A03	44,868,710	65,694	1464.14	17,432	388.51
A04	25,679,024	32,503	1265.74	7375	287.20
A05	45,991,561	65,210	1417.87	14,254	309.93
A06	48,704,706	50,312	1033.00	12,939	265.66
A07	32,302,721	28,853	893.21	7210	223.20
A08	28,329,074	14,327	505.73	4121	145.47
A09	65,862,748	59,432	902.36	14,261	216.53
A10	26,592,803	27,124	1019.98	6928	260.52
C01	57,880,920	10,932	188.87	2535	43.80
C02	65,293,782	7886	120.78	868	13.29
C03	79,061,710	45,628	577.12	9611	121.56
C04	71,179,181	88,569	1244.31	14,848	208.60
C05	59,550,008	25,467	427.66	6015	101.01
C06	52,512,057	26,060	496.27	5506	104.85
C07	60,986,212	34,183	560.50	6204	101.73
C08	53,660,391	34,087	635.24	6011	112.02
C09	68,416,614	45,121	659.50	7527	110.02
Whole	960,820,604	752,940	783.64	165,381	172.12

Note: Chr: chromosome number; Length: chromosome length; No. of SNPs: number of SNPs; SNP Density: number of SNPs per Mb; No. of InDels: number of InDels; InDel Density: number of InDels per Mb.

**Table 5:** Statistical table of candidate interval information.

Chr	Start (Mb)	End (Mb)	Length (Mb)	No. of Genes	No. of SNPs	No. of InDels	No. of Genes with LEVs
A03	19.30	22.19	2.89	473	6311	1808	124

Note: Ch: chromosome where the candidate interval is located; Start: start position of the candidate interval; End: end position of the candidate interval; Length: length of the candidate interval; No. of Genes: total number of genes in the candidate interval; No. of Genes (transcripts) with LEVs: number of genes containing large–effect variants (LEVs: large–effect variants; variants that lead to changes in protein sequences) in the candidate interval.

**Table 6:** Predicted genes and their putative functions within the mapped region.

ZS11 Gene ID	Genomic Position	Function
BnaA03G0378200ZS	A03:20232019–20232411	––
BnaA03G0378500ZS	A03:20251212–20253333	Beta–amylase 1, chloroplastic
BnaA03G0378800ZS	A03:20263423–20264925	Putative ammonium transporter 1 member 5
BnaA03G0379000ZS	A03:20291126–20293345	Glutamyl–tRNA(Gln) amidotransferase subunit B, chloroplastic/mitochondrial
BnaA03G0379100ZS	A03:20293607–20300023	Vesicle transport protein SEC20
BnaA03G0379200ZS	A03:20301123–20304488	SNF2 domain–containing protein CLASSY 4
BnaA03G0380500ZS	A03:20423757–20424182	––
BnaA03G0381200ZS	A03:20463970–20468450	Dual–specificity protein kinase splA
BnaA03G0382800ZS	A03:20573770–20575946	Long–chain acyl–CoA synthetase 8
BnaA03G0384600ZS	A03:20699253–20700104	Long–chain acyl–CoA synthetase 8
BnaA03G0384700ZS	A03:20700666–20702921	Indole–3–glycerol phosphate synthase, chloroplastic
BnaA03G0384800ZS	A03:20704164–20705354	Kelch repeat–containing protein At1g19470
BnaA03G0385100ZS	A03:20731616–20732260	––
BnaA03G0385300ZS	A03:20734246–20735776	Ribonuclease Z, chloroplastic
BnaA03G0385400ZS	A03:20735919–20738394	3–oxoacyl–[acyl–carrier–protein] synthase, mitochondrial
BnaA03G0385600ZS	A03:20743302–20746466	GDSL esterase/lipase At2g04570
BnaA03G0385700ZS	A03:20750637–20751722	Cysteine–rich receptor–like protein kinase
BnaA03G0385800ZS	A03:20755382–20758254	BTB/POZ domain–containing protein
BnaA03G0385900ZS	A03:20764333–20765103	Fasciclin–like arabinogalactan protein 7
BnaA03G0386100ZS	A03:20780894–20783666	Threonine––tRNA ligase, chloroplastic/mitochondrial
BnaA03G0386200ZS	A03:20784219–20785820	N–acetyltransferase 9–like protein
BnaA03G0386300ZS	A03:20786172–20787868	Cytochrome b561 and DOMON domain–containing protein
BnaA03G0386400ZS	A03:20788373–20790448	Pentatricopeptide repeat–containing protein
BnaA03G0386600ZS	A03:20801601–20801915	––
BnaA03G0386800ZS	A03:20805347–20808022	WRKY transcription factor 1
BnaA03G0386900ZS	A03:20808146–20813983	Histidine kinase
BnaA03G0387000ZS	A03:20816714–20819484	Altered inheritance rate of mitochondria protein 25
BnaA03G0387100ZS	A03:20822473–20823481	––
BnaA03G0387200ZS	A03:20824629–20825512	Chlorophyll a–b binding protein 151, chloroplastic
BnaA03G0387300ZS	A03:20826103–20831087	Nuclear pore complex protein NUP133
BnaA03G0387900ZS	A03:20867173–20868055	––
BnaA03G0388100ZS	A03:20876795–20877452	Protein PROTON GRADIENT REGULATION 5, chloroplastic
BnaA03G0388500ZS	A03:20891600–20894545	Nucleobase–ascorbate transporter 1
BnaA03G0388600ZS	A03:20897334–20901330	DNA replication licensing factor MCM5
BnaA03G0388700ZS	A03:20902447–20906312	ATPase 6, plasma membrane–type
BnaA03G0388800ZS	A03:20908432–20913818	Cycloartenol synthase
BnaA03G0388900ZS	A03:20915798–20918872	U–box domain–containing protein 34
BnaA03G0389100ZS	A03:20921900–20922723	––
BnaA03G0389300ZS	A03:20934183–20935372	Vacuolar protein sorting–associated protein 2 homolog 1
BnaA03G0389400ZS	A03:20937985–20944590	Probable UDP–N–acetylglucosamine––peptide N–acetylglucosaminyltransferase SEC
BnaA03G0389500ZS	A03:20945439–20948174	12–oxophytodienoate reductase 3
BnaA03G0389600ZS	A03:20949288–20951130	Enoyl–[acyl–carrier–protein] reductase [NADH], chloroplastic

## Data Availability

Data are contained within the article.

## Supplementary Materials

The following supporting information can be downloaded at: https://www.mdpi.com/article/10.3390/plants13162298/s1, Figure S1: Chlorophyll leaching from leaves; Table S1: SSR markers information; Table S2: Length and location information of PCR-amplified fragments of 12 SSR markers; Table S3: Information about 42 genes with large effect variants (LEVs); Table S4: The relative expression of 42 genes in dwarf and high individuals; Table S5: The qRT -PCR primer of 42 genes.
