# Supplementary material for: Identification of Dwarfing Candidate Genes in Brassica napus L. LSW2018 through BSA–Seq and Genetic Mapping
Source: Plants (Basel). 2024 Aug 18;13(16):2298. doi: 10.3390/plants13162298 (PMC11359780; doi:10.3390/plants13162298)
Supplement: Supplementary file 1 [file plants-13-02298-s001.zip › Figure S1. Chlorophyll leaching from leaves.pdf]

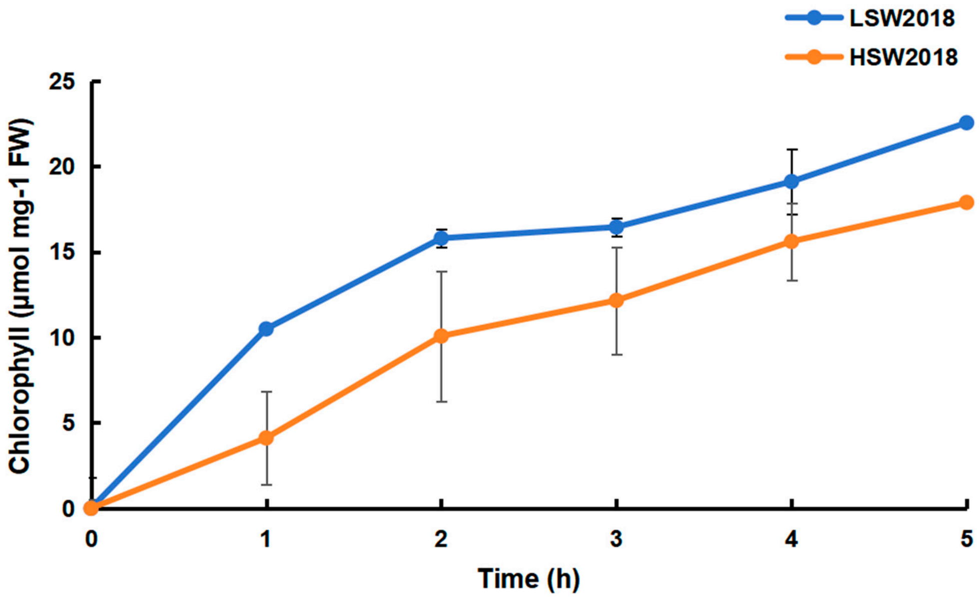

Figure S1. Chlorophyll leaching from leaves.

Not:Leaves were submerged in 80% ethanol and aliquot were taken at the given time points to assess the chlorophyll concentration of the ethanol. Results shown are means from three replicates [±standard deviation (SD)].
