# Supplementary material for: Identification of Dwarfing Candidate Genes in Brassica napus L. LSW2018 through BSA–Seq and Genetic Mapping
Source: Plants (Basel). 2024 Aug 18;13(16):2298. doi: 10.3390/plants13162298 (PMC11359780; doi:10.3390/plants13162298)
Supplement: Supplementary file 1 [file plants-13-02298-s001.zip › Table S1. SSR markers information.pdf]

Table S1. SSR markers information

No.	Name	Forward primer（5′ - 3′ ）	Reverse primer（5′ - 3′ ）
1	PA01	ACACAGAAGAAAAGTTTCTGAAGAGTA	ATGACATCAGTGAGAAGCTAGTGC
2	PA04	GCCACCACCGAAGAGGACA	GCTTGATTCCGTCTCTTGCCA
3	PA06	TGCAGGTTATCTGATTGCTGA	ACGCCTTCAACATTATTAACTCTTTGC
4	PA08	TGCTACCTCTCTTTCCCTGT	TTCCATCGCTGCTTTCTGATGA
5	PA10	TGGTACTTGATCAAATCAAAGCATATCTAAC	ATACTAGTAGTTAATTCCATTTGCGTGAC
6	PA12	TCGATCGATGTCGGAAAGGGT	CGGTTTCACACACATCTGGCT
7	PA14	GCCATTGTTTTCGGTCGCTT	CTCTGAGACTCAACCTTGGTTAAAGAT
8	PA16	TTTGTAGAGAATCTCACGTTTCGGA	CCGCTGCAACGCAGAGC
9	PA18	TTGCTGCCTAATCTTTCCCGTT	ACTGGAGGCTTTTGGTTCTTTGT
10	PA20	CTTGCACGTGCCGACGA	TTCTCCTCCATGAACCTTTGGCT
11	PA22	GACGAACTGTACGGGTCAATCT	TGAGCAACTATCTTGAATATCTTCTCTTCT
12	PA24	ATAAACAGTCCGTAATGCCTTCCT	GCGGTGCATGGGGGTTG
13	PA26	TCCAGCACTAAGACTAGTACCAACA	TGCTTCTTTGGGAGAGGAGGC
14	PA28	CCCCCTGTTAATTCAGTAGCTTATTCA	TTCAAAATGCCAATCATAGATTTGTTTTCA
15	PA31	TGTCATAGAGCTTACGTTGTCTTCA	CCCTGCTGTTTTAGGTGTGGT
16	PA33	AAGGCGTCATTGAGTTTCTGGT	AGGAAGAGGAATGTTAGAGTCAACG
17	PA36	ACCACGTCTAACGTAAGCTGGATT	AGATTGTTGTGAGTTTTACACTTCCGT
18	PA38	TCTTTGTCAGCTTGTTGGCGT	CGGCCAGCAGTTGCGTC
19	PA39	ACCAAGAAACCAAGGAACAATGC	ACCATTAGTGAACATTGTATTCGTTTGA
20	PA41	CACATCTGGAGAAGGCCAAGGA	TCCAAATTGGTAATAACGAGAAGAGAGA
21	PA42	GCACCTCCTAAATAATCAATAGAGAAACAA	CCCTCCTTAGCCATTTTCTCCCA
22	PA44	TGCAATAATTTCGGTTACCTTCGGT	TTTCGGTTCGGTGGTTTCGT
23	PA45	GGACAAGTTCACTAAACAACTAAACTATGA	ACATACGAGGTGAACGCCAC
24	PA47	AGGATTGGTTTGCTTTGGAAGTAGC	CCGGAGGGATTCAGCAGCA
25	PA48	TGCAAAACAGTATAACATAATCAATCAGAACA	CCATCTCTGTCACTGATGTGTGG
26	PA49	TCTCCTTCCGAACAACCTTAGC	ACTGTAAGGGTACATGGAGTCTGT
27	PA50	ACGACACCATGATACAAAATTCAACA	GGCAGATGAATGAATAAGATGCGTT
28	PA52	TCTGACGGAGAGAAAATAGTGGAAAT	TGGGGTTGACCGGTATGGT
29	PA53	GCTGACTAACAACTCAACACTCGT	GCCTCAGCATCACCTTTAGGC
30	PA55	TGGAACTTCATCAAACTCCTCCGA	GCCATTCCCTGGACCAAAAACA
