# Supplementary material for: Identification of Dwarfing Candidate Genes in Brassica napus L. LSW2018 through BSA–Seq and Genetic Mapping
Source: Plants (Basel). 2024 Aug 18;13(16):2298. doi: 10.3390/plants13162298 (PMC11359780; doi:10.3390/plants13162298)
Supplement: Supplementary file 1 [file plants-13-02298-s001.zip › Table S2 Length and location information of PCR-amplified fragments of 12 SSR markers.pdf]

Table S2. Length and location information of PCR-amplified fragments of 12 SSR markers

SSR markers	PCR product length（bp）	PCR product situation
PA04	130	A03_+_19459935_19460064
PA10	85	A03_+_19959002_19959086
PA12	115	A03_+_20025321_20025403
PA14	130	A03_+_20068569_20068698
PA16	126	A03_+_20069862_20069947
PA20	101	A03_+_20141508_20141608
PA24	100	A03_+_20233254_20233350
PA38	93	A03_+_20926470_20926562
PA41	80	A03_+_20942174_20942253
PA47	105	A03_+_20997116_20997220
PA49	98	A03_+_21018718_21018815
PA53	99	A03_+_21788154_21788250_0
