# Supplementary material for: Identification of Dwarfing Candidate Genes in Brassica napus L. LSW2018 through BSA–Seq and Genetic Mapping
Source: Plants (Basel). 2024 Aug 18;13(16):2298. doi: 10.3390/plants13162298 (PMC11359780; doi:10.3390/plants13162298)
Supplement: Supplementary file 1 [file plants-13-02298-s001.zip › Table S3 Information about 42 genes with large effect variants (LEVs).pdf]

Table S3. Information about 42 genes with large effect variants (LEVs)

ID	Chr	Pos	Ref	Alt	Annotation-effect	Annotation-effect detail
BnaA03G0378200ZS	A03	20232058	CTGG	C	nonframeshift deletion	BnaA03G0378200ZS:BnaA03T0378200ZS:exon1:c.41_43del:p.14_15del,
BnaA03G0378200ZS	A03	20232052	G	GTGC	nonframeshift insertion	BnaA03G0378200ZS:BnaA03T0378200ZS:exon1:c.34_35insTGC:p.V12delinsVL,
BnaA03G0378500ZS	A03	20251869	C	G	nonsynonymous SNV	BnaA03G0378500ZS:BnaA03T0378500ZS:exon4:c.G1011C:p.E337D,
BnaA03G0378500ZS	A03	20251899	T	A	nonsynonymous SNV	BnaA03G0378500ZS:BnaA03T0378500ZS:exon4:c.A981T:p.E327D,
BnaA03G0378500ZS	A03	20252524	G	T	nonsynonymous SNV	BnaA03G0378500ZS:BnaA03T0378500ZS:exon2:c.C563A:p.A188E,
BnaA03G0378500ZS	A03	20252870	T	A	nonsynonymous SNV	BnaA03G0378500ZS:BnaA03T0378500ZS:exon1:c.A464T:p.K155M,
BnaA03G0378800ZS	A03	20263720	GCTGGCT	G	nonframeshift deletion	BnaA03G0378800ZS:BnaA03T0378800ZS:exon1:c.1200_1205del:p.400_402del,
BnaA03G0379000ZS	A03	20292024	C	G	nonsynonymous SNV	BnaA03G0379000ZS:BnaA03T0379000ZS:exon2:c.C820G:p.P274A,
BnaA03G0379000ZS	A03	20293342	G	GTGA	stopgain	BnaA03G0379000ZS:BnaA03T0379000ZS:exon8:c.1539_1540insTGA:p.E513delinsEX,
BnaA03G0379100ZS	A03	20299533	GCTTAACAACC	G	frameshift deletion	BnaA03G0379100ZS:BnaA03T0379100ZS:exon2:c.296_299del:p.W99fs,
BnaA03G0379100ZS	A03	20299447	G	GAAC	nonframeshift insertion	BnaA03G0379100ZS:BnaA03T0379100ZS:exon3:c.322_323insGTT:p.T108delinsSS,
BnaA03G0379100ZS	A03	20299837	G	A	nonsynonymous SNV	BnaA03G0379100ZS:BnaA03T0379100ZS:exon1:c.C187T:p.P63S,
BnaA03G0379100ZS	A03	20299899	A	T	nonsynonymous SNV	BnaA03G0379100ZS:BnaA03T0379100ZS:exon1:c.T125A:p.I42N,

Table S3. Cont.

ID	Chr	Pos	Ref	Alt	Annotation-effect	Annotation-effect detail
BnaA03G0379200ZS	A03	20301825	C	G	nonsynonymous SNV	BnaA03G0379200ZS:BnaA03T0379200ZS:exon4:c.G2292C:p.K764N,
BnaA03G0379200ZS	A03	20302483	G	A	nonsynonymous SNV	BnaA03G0379200ZS:BnaA03T0379200ZS:exon4:c.C1634T:p.S545L,
BnaA03G0379200ZS	A03	20302741	T	C	nonsynonymous SNV	BnaA03G0379200ZS:BnaA03T0379200ZS:exon4:c.A1376G:p.D459G,
BnaA03G0379200ZS	A03	20302792	T	C	nonsynonymous SNV	BnaA03G0379200ZS:BnaA03T0379200ZS:exon4:c.A1325G:p.Q442R,
BnaA03G0379200ZS	A03	20303114	T	C	nonsynonymous SNV	BnaA03G0379200ZS:BnaA03T0379200ZS:exon3:c.A1079G:p.Y360C,
BnaA03G0379200ZS	A03	20303435	A	T	nonsynonymous SNV	BnaA03G0379200ZS:BnaA03T0379200ZS:exon2:c.T850A:p.Y284N,
BnaA03G0379200ZS	A03	20303488	G	A	nonsynonymous SNV	BnaA03G0379200ZS:BnaA03T0379200ZS:exon2:c.C797T:p.S266L,
BnaA03G0379200ZS	A03	20303491	C	T	nonsynonymous SNV	BnaA03G0379200ZS:BnaA03T0379200ZS:exon2:c.G794A:p.R265H,
BnaA03G0379200ZS	A03	20303500	C	T	nonsynonymous SNV	BnaA03G0379200ZS:BnaA03T0379200ZS:exon2:c.G785A:p.R262K,
BnaA03G0379200ZS	A03	20303667	A	C	nonsynonymous SNV	BnaA03G0379200ZS:BnaA03T0379200ZS:exon2:c.T618G:p.D206E,
BnaA03G0379200ZS	A03	20303683	T	C	nonsynonymous SNV	BnaA03G0379200ZS:BnaA03T0379200ZS:exon2:c.A602G:p.D201G,
BnaA03G0379200ZS	A03	20304107	C	T	nonsynonymous SNV	BnaA03G0379200ZS:BnaA03T0379200ZS:exon1:c.G382A:p.E128K,
BnaA03G0380500ZS	A03	20424088	G	A	nonsynonymous SNV	BnaA03G0380500ZS:BnaA03T0380500ZS:exon1:c.C95T:p.S32F,

Table S3. Cont.

ID	Chr	Pos	Ref	Alt	Annotation-effect	Annotation-effect detail
BnaA03G0381200ZS	A03	20464610	A	G	nonsynonymous SNV	BnaA03G0381200ZS:BnaA03T0381200ZS:exon2:c.A10G:p.T4A,
BnaA03G0381200ZS	A03	20465496	A	T	nonsynonymous SNV	BnaA03G0381200ZS:BnaA03T0381200ZS:exon2:c.A896T:p.K299M,
BnaA03G0381200ZS	A03	20466136	G	T	nonsynonymous SNV	BnaA03G0381200ZS:BnaA03T0381200ZS:exon2:c.G1536T:p.M512I,
BnaA03G0381200ZS	A03	20466146	G	A	nonsynonymous SNV	BnaA03G0381200ZS:BnaA03T0381200ZS:exon2:c.G1546A:p.D516N,
BnaA03G0381200ZS	A03	20466279	G	C	nonsynonymous SNV	BnaA03G0381200ZS:BnaA03T0381200ZS:exon2:c.G1679C:p.R560T,
BnaA03G0382800ZS	A03	20575874	C	T	nonsynonymous SNV	BnaA03G0382800ZS:BnaA03T0382800ZS:exon1:c.G73A:p.V25I,
BnaA03G0382800ZS	A03	20575908	A	C	nonsynonymous SNV	BnaA03G0382800ZS:BnaA03T0382800ZS:exon1:c.T39G:p.N13K,
BnaA03G0384600ZS	A03	20699949	A	G	nonsynonymous SNV	BnaA03G0384600ZS:BnaA03T0384600ZS:exon3:c.A184G:p.T62A,
BnaA03G0384700ZS	A03	20702229	A	T	nonsynonymous SNV	BnaA03G0384700ZS:BnaA03T0384700ZS:exon6:c.A871T:p.M291L,
BnaA03G0384800ZS	A03	20704531	T	A	nonsynonymous SNV	BnaA03G0384800ZS:BnaA03T0384800ZS:exon2:c.A725T:p.E242V,
BnaA03G0385100ZS	A03	20732015	AAAG	A	nonframeshift deletion	BnaA03G0385100ZS:BnaA03T0385100ZS:exon1:c.401_403del:p.134_135del,
BnaA03G0385100ZS	A03	20732129	CATGATG	C	nonframeshift deletion	BnaA03G0385100ZS:BnaA03T0385100ZS:exon1:c.515_520del:p.172_174del,
BnaA03G0385100ZS	A03	20731816	C	CAGT	nonframeshift insertion	BnaA03G0385100ZS:BnaA03T0385100ZS:exon1:c.201_202insAGT:p.G67delinsGS,

Table S3. Cont.

ID	Chr	Pos	Ref	Alt	Annotation-effect	Annotation-effect detail
BnaA03G0385100ZS	A03	20731840	C	A	nonsynonymous SNV	BnaA03G0385100ZS:BnaA03T0385100ZS:exon1:c.C225A:p.D75E,
BnaA03G0385100ZS	A03	20731874	G	T	nonsynonymous SNV	BnaA03G0385100ZS:BnaA03T0385100ZS:exon1:c.G259T:p.A87S,
BnaA03G0385100ZS	A03	20732013	A	C	nonsynonymous SNV	BnaA03G0385100ZS:BnaA03T0385100ZS:exon1:c.A398C:p.Y133S,
BnaA03G0385100ZS	A03	20732076	C	T	nonsynonymous SNV	BnaA03G0385100ZS:BnaA03T0385100ZS:exon1:c.C461T:p.A154V,
BnaA03G0385100ZS	A03	20732159	G	A	nonsynonymous SNV	BnaA03G0385100ZS:BnaA03T0385100ZS:exon1:c.G544A:p.D182N,
BnaA03G0385100ZS	A03	20732164	C	A	nonsynonymous SNV	BnaA03G0385100ZS:BnaA03T0385100ZS:exon1:c.C549A:p.F183L,
BnaA03G0385100ZS	A03	20732211	A	G	nonsynonymous SNV	BnaA03G0385100ZS:BnaA03T0385100ZS:exon1:c.A596G:p.K199R,
BnaA03G0385300ZS	A03	20734252	A	C	nonsynonymous SNV	BnaA03G0385300ZS:BnaA03T0385300ZS:exon1:c.A7C:p.M3L,
BnaA03G0385300ZS	A03	20734254	G	C	nonsynonymous SNV	BnaA03G0385300ZS:BnaA03T0385300ZS:exon1:c.G9C:p.M3I,
BnaA03G0385300ZS	A03	20734842	T	C	nonsynonymous SNV	BnaA03G0385300ZS:BnaA03T0385300ZS:exon2:c.T521C:p.V174A,
BnaA03G0385300ZS	A03	20735296	G	A	nonsynonymous SNV	BnaA03G0385300ZS:BnaA03T0385300ZS:exon4:c.G811A:p.V271M,
BnaA03G0385400ZS	A03	20738308	G	GGAA	nonframeshift insertion	BnaA03G0385400ZS:BnaA03T0385400ZS:exon1:c.86_87insTTC:p.S29delinsSS,
BnaA03G0385400ZS	A03	20736024	C	A	nonsynonymous SNV	BnaA03G0385400ZS:BnaA03T0385400ZS:exon14:c.G1284T:p.K428N,

Table S3. Cont.

ID	Chr	Pos	Ref	Alt	Annotation-effect	Annotation-effect detail
BnaA03G0385400ZS	A03	20736025	T	C	nonsynonymous SNV	BnaA03G0385400ZS:BnaA03T0385400ZS:exon14:c.A1283G:p.K428R,
BnaA03G0385400ZS	A03	20738028	G	A	nonsynonymous SNV	BnaA03G0385400ZS:BnaA03T0385400ZS:exon2:c.C292T:p.H98Y,
BnaA03G0385600ZS	A03	20743343	G	T	nonsynonymous SNV	BnaA03G0385600ZS:BnaA03T0385600ZS:exon1:c.G5T:p.G2V,
BnaA03G0385700ZS	A03	20750688	G	A	nonsynonymous SNV	BnaA03G0385700ZS:BnaA03T0385700ZS:exon1:c.G52A:p.V18I,
BnaA03G0385700ZS	A03	20750742	A	G	nonsynonymous SNV	BnaA03G0385700ZS:BnaA03T0385700ZS:exon1:c.A106G:p.R36G,
BnaA03G0385700ZS	A03	20750768	G	C	nonsynonymous SNV	BnaA03G0385700ZS:BnaA03T0385700ZS:exon1:c.G132C:p.M44I,
BnaA03G0385700ZS	A03	20750787	A	G	nonsynonymous SNV	BnaA03G0385700ZS:BnaA03T0385700ZS:exon1:c.A151G:p.S51G,
BnaA03G0385700ZS	A03	20750788	G	A	nonsynonymous SNV	BnaA03G0385700ZS:BnaA03T0385700ZS:exon1:c.G152A:p.S51N,
BnaA03G0385700ZS	A03	20750885	G	T	nonsynonymous SNV	BnaA03G0385700ZS:BnaA03T0385700ZS:exon1:c.G249T:p.E83D,
BnaA03G0385700ZS	A03	20750906	A	T	nonsynonymous SNV	BnaA03G0385700ZS:BnaA03T0385700ZS:exon1:c.A270T:p.E90D,
BnaA03G0385700ZS	A03	20750909	G	T	nonsynonymous SNV	BnaA03G0385700ZS:BnaA03T0385700ZS:exon1:c.G273T:p.Q91H,
BnaA03G0385700ZS	A03	20750920	A	T	nonsynonymous SNV	BnaA03G0385700ZS:BnaA03T0385700ZS:exon1:c.A284T:p.E95V,
BnaA03G0385700ZS	A03	20750922	G	T	nonsynonymous SNV	BnaA03G0385700ZS:BnaA03T0385700ZS:exon1:c.G286T:p.V96L,

Table S3. Cont.

ID	Chr	Pos	Ref	Alt	Annotation-effect	Annotation-effect detail
BnaA03G0385700ZS	A03	20750967	C	G	nonsynonymous SNV	BnaA03G0385700ZS:BnaA03T0385700ZS:exon1:c.C331G:p.Q111E,
BnaA03G0385700ZS	A03	20751003	A	C	nonsynonymous SNV	BnaA03G0385700ZS:BnaA03T0385700ZS:exon1:c.A367C:p.S123R,
BnaA03G0385800ZS	A03	20755567	C	T	nonsynonymous SNV	BnaA03G0385800ZS:BnaA03T0385800ZS:exon1:c.C85T:p.P29S,BnaA03G0385800ZS:BnaA03T0385800ZS.1:exon1:c.C85T:p.P29S,
BnaA03G0385800ZS	A03	20755654	A	T	nonsynonymous SNV	BnaA03G0385800ZS:BnaA03T0385800ZS:exon1:c.A172T:p.T58S,BnaA03G0385800ZS:BnaA03T0385800ZS.1:exon1:c.A172T:p.T58S,
BnaA03G0385800ZS	A03	20756777	C	A	nonsynonymous SNV	BnaA03G0385800ZS:BnaA03T0385800ZS:exon2:c.C618A:p.D206E,BnaA03G0385800ZS:BnaA03T0385800ZS.1:exon2:c.C612A:p.D204E,
BnaA03G0385800ZS	A03	20757202	A	C	nonsynonymous SNV	BnaA03G0385800ZS:BnaA03T0385800ZS:exon2:c.A1043C:p.N348T,BnaA03G0385800ZS:BnaA03T0385800ZS.1:exon2:c.A1037C:p.N346T,
BnaA03G0385800ZS	A03	20758023	T	C	nonsynonymous SNV	BnaA03G0385800ZS:BnaA03T0385800ZS:exon5:c.T1619C:p.L540S,BnaA03G0385800ZS:BnaA03T0385800ZS.1:exon5:c.T1613C:p.L538S,
BnaA03G0385900ZS	A03	20765055	T	A	nonsynonymous SNV	BnaA03G0385900ZS:BnaA03T0385900ZS:exon1:c.T723A:p.F241L,
BnaA03G0386100ZS	A03	20783566	A	AGGGTTT	nonframeshift insertion	BnaA03G0386100ZS:BnaA03T0386100ZS:exon1:c.30_31insAAACCC:p.S11delinsKPS,
BnaA03G0386100ZS	A03	20783570	G	GAGGAAGGAAGAGGAA	nonframeshift insertion	BnaA03G0386100ZS:BnaA03T0386100ZS:exon1:c.26_27insTTCCTCTTCCTTCCT:p.L9delinsLSSSFL,

Table S3. Cont.

ID	Chr	Pos	Ref	Alt	Annotation-effect	Annotation-effect detail
BnaA03G0386100ZS	A03	20782238	C	T	nonsynonymous SNV	BnaA03G0386100ZS:BnaA03T0386100ZS:exon4:c.G956A:p.R319K,
BnaA03G0386100ZS	A03	20782817	C	T	nonsynonymous SNV	BnaA03G0386100ZS:BnaA03T0386100ZS:exon3:c.G473A:p.R158K,
BnaA03G0386100ZS	A03	20783550	C	T	nonsynonymous SNV	BnaA03G0386100ZS:BnaA03T0386100ZS:exon1:c.G47A:p.R16K,
BnaA03G0386200ZS	A03	20784270	T	A	nonsynonymous SNV	BnaA03G0386200ZS:BnaA03T0386200ZS:exon6:c.A612T:p.Q204H,
BnaA03G0386300ZS	A03	20786175	T	G	nonsynonymous SNV	BnaA03G0386300ZS:BnaA03T0386300ZS:exon1:c.T4G:p.S2A,
BnaA03G0386300ZS	A03	20786727	G	A	nonsynonymous SNV	BnaA03G0386300ZS:BnaA03T0386300ZS:exon1:c.G556A:p.A186T,
BnaA03G0386300ZS	A03	20787276	G	A	nonsynonymous SNV	BnaA03G0386300ZS:BnaA03T0386300ZS:exon2:c.G620A:p.R207K,
BnaA03G0386400ZS	A03	20788610	CTT	C	frameshift deletion	BnaA03G0386400ZS:BnaA03T0386400ZS:exon1:c.1837_1838del:p.K613fs,
BnaA03G0386400ZS	A03	20788458	T	C	nonsynonymous SNV	BnaA03G0386400ZS:BnaA03T0386400ZS:exon1:c.A1991G:p.E664G,
BnaA03G0386400ZS	A03	20788639	C	A	nonsynonymous SNV	BnaA03G0386400ZS:BnaA03T0386400ZS:exon1:c.G1810T:p.V604L,
BnaA03G0386400ZS	A03	20788709	T	C	nonsynonymous SNV	BnaA03G0386400ZS:BnaA03T0386400ZS:exon1:c.A1740G:p.I580M,
BnaA03G0386400ZS	A03	20788815	T	C	nonsynonymous SNV	BnaA03G0386400ZS:BnaA03T0386400ZS:exon1:c.A1634G:p.K545R,
BnaA03G0386400ZS	A03	20788839	A	G	nonsynonymous SNV	BnaA03G0386400ZS:BnaA03T0386400ZS:exon1:c.T1610C:p.L537S,

Table S3. Cont.

ID	Chr	Pos	Ref	Alt	Annotation-effect	Annotation-effect detail
BnaA03G0386400ZS	A03	20789236	T	G	nonsynonymous SNV	BnaA03G0386400ZS:BnaA03T0386400ZS:exon1:c.A1213C:p.M405L,
BnaA03G0386600ZS	A03	20801911	A	T	nonsynonymous SNV	BnaA03G0386600ZS:BnaA03T0386600ZS:exon1:c.T5A:p.L2H,
BnaA03G0386800ZS	A03	20806209	G	C	nonsynonymous SNV	BnaA03G0386800ZS:BnaA03T0386800ZS:exon2:c.G242C:p.S81T,
BnaA03G0386900ZS	A03	20808396	G	T	nonsynonymous SNV	BnaA03G0386900ZS:BnaA03T0386900ZS:exon11:c.C3363A:p.D1121E,
BnaA03G0386900ZS	A03	20809067	G	T	nonsynonymous SNV	BnaA03G0386900ZS:BnaA03T0386900ZS:exon10:c.C2774A:p.P925H,
BnaA03G0386900ZS	A03	20809941	G	A	nonsynonymous SNV	BnaA03G0386900ZS:BnaA03T0386900ZS:exon9:c.C2159T:p.S720L,
BnaA03G0386900ZS	A03	20813211	T	C	nonsynonymous SNV	BnaA03G0386900ZS:BnaA03T0386900ZS:exon2:c.A308G:p.D103G,
BnaA03G0386900ZS	A03	20813423	T	G	nonsynonymous SNV	BnaA03G0386900ZS:BnaA03T0386900ZS:exon1:c.A176C:p.D59A,
BnaA03G0386900ZS	A03	20813427	A	C	nonsynonymous SNV	BnaA03G0386900ZS:BnaA03T0386900ZS:exon1:c.T172G:p.S58A,
BnaA03G0386900ZS	A03	20808534	C	T	stopgain	BnaA03G0386900ZS:BnaA03T0386900ZS:exon11:c.G3225A:p.W1075X,
BnaA03G0387000ZS	A03	20817296	T	C	nonsynonymous SNV	BnaA03G0387000ZS:BnaA03T0387000ZS:exon9:c.A892G:p.M298V,
BnaA03G0387000ZS	A03	20819071	A	T	nonsynonymous SNV	BnaA03G0387000ZS:BnaA03T0387000ZS:exon2:c.T209A:p.V70D,
BnaA03G0387000ZS	A03	20819078	A	G	nonsynonymous SNV	BnaA03G0387000ZS:BnaA03T0387000ZS:exon2:c.T202C:p.F68L,

Table S3. Cont.

ID	Chr	Pos	Ref	Alt	Annotation-effect	Annotation-effect detail
BnaA03G0387000ZS	A03	20819080	C	G	nonsynonymous SNV	BnaA03G0387000ZS:BnaA03T0387000ZS:exon2:c.G200C:p.S67T,
BnaA03G0387000ZS	A03	20819471	A	T	nonsynonymous SNV	BnaA03G0387000ZS:BnaA03T0387000ZS:exon1:c.T14A:p.L5H,
BnaA03G0387100ZS	A03	20823391	ACAT	A	nonframeshift deletion	BnaA03G0387100ZS:BnaA03T0387100ZS:exon4:c.643_645del:p.215_215del,
BnaA03G0387100ZS	A03	20823026	A	G	nonsynonymous SNV	BnaA03G0387100ZS:BnaA03T0387100ZS:exon4:c.A277G:p.N93D,
BnaA03G0387100ZS	A03	20823436	T	A	nonsynonymous SNV	BnaA03G0387100ZS:BnaA03T0387100ZS:exon4:c.T687A:p.S229R,
BnaA03G0387200ZS	A03	20825424	G	A	nonsynonymous SNV	BnaA03G0387200ZS:BnaA03T0387200ZS:exon1:c.C89T:p.S30F,
BnaA03G0387300ZS	A03	20826548	A	G	nonsynonymous SNV	BnaA03G0387300ZS:BnaA03T0387300ZS:exon8:c.T3662C:p.V1221A,BnaA03G0387300ZS:BnaA03T0387300ZS.1:exon8:c.T3644C:p.V1215A,
BnaA03G0387300ZS	A03	20826600	T	C	nonsynonymous SNV	BnaA03G0387300ZS:BnaA03T0387300ZS:exon8:c.A3610G:p.K1204E,BnaA03G0387300ZS:BnaA03T0387300ZS.1:exon8:c.A3592G:p.K1198E,
BnaA03G0387300ZS	A03	20826734	C	A	nonsynonymous SNV	BnaA03G0387300ZS:BnaA03T0387300ZS:exon8:c.G3476T:p.C1159F,BnaA03G0387300ZS:BnaA03T0387300ZS.1:exon8:c.G3458T:p.C1153F,
BnaA03G0387300ZS	A03	20826933	G	A	nonsynonymous SNV	BnaA03G0387300ZS:BnaA03T0387300ZS.2:exon7:c.C3355T:p.L1119F,

Table S3. Cont.

ID	Chr	Pos	Ref	Alt	Annotation-effect	Annotation-effect detail
BnaA03G0387300ZS	A03	20827676	T	C	nonsynonymous SNV	BnaA03G0387300ZS:BnaA03T0387300ZS:exon5:c.A2882G:p.K961R,BnaA03G0387300ZS:BnaA03T0387300ZS.1:exon5:c.A2864G:p.K955R,BnaA03G0387300ZS:BnaA03T0387300ZS.2:exon5:c.A2882G:p.K961R,
BnaA03G0387300ZS	A03	20827882	T	A	nonsynonymous SNV	BnaA03G0387300ZS:BnaA03T0387300ZS:exon4:c.A2757T:p.L919F,BnaA03G0387300ZS:BnaA03T0387300ZS.1:exon4:c.A2739T:p.L913F,BnaA03G0387300ZS:BnaA03T0387300ZS.2:exon4:c.A2757T:p.L919F,
BnaA03G0387300ZS	A03	20829297	G	C	nonsynonymous SNV	BnaA03G0387300ZS:BnaA03T0387300ZS:exon3:c.C1558G:p.L520V,BnaA03G0387300ZS:BnaA03T0387300ZS.1:exon3:c.C1540G:p.L514V,BnaA03G0387300ZS:BnaA03T0387300ZS.2:exon3:c.C1558G:p.L520V,
BnaA03G0387300ZS	A03	20829510	T	G	nonsynonymous SNV	BnaA03G0387300ZS:BnaA03T0387300ZS:exon3:c.A1345C:p.K449Q,BnaA03G0387300ZS:BnaA03T0387300ZS.1:exon3:c.A1327C:p.K443Q,BnaA03G0387300ZS:BnaA03T0387300ZS.2:exon3:c.A1345C:p.K449Q,
BnaA03G0387300ZS	A03	20829822	T	C	nonsynonymous SNV	BnaA03G0387300ZS:BnaA03T0387300ZS:exon3:c.A1033G:p.N345D,BnaA03G0387300ZS:BnaA03T0387300ZS.1:exon3:c.A1015G:p.N339D,BnaA03G0387300ZS:BnaA03T0387300ZS.2:exon3:c.A1033G:p.N345D,

Table S3. Cont.

ID	Chr	Pos	Ref	Alt	Annotation-effect	Annotation-effect detail
BnaA03G0387300ZS	A03	20830025	T	G	nonsynonymous SNV	BnaA03G0387300ZS:BnaA03T0387300ZS:exon3:c.A830C:p.K277T,BnaA03G0387300ZS:BnaA03T0387300ZS.1:exon3:c.A812C:p.K271T,BnaA03G0387300ZS:BnaA03T0387300ZS.2:exon3:c.A830C:p.K277T,
BnaA03G0387300ZS	A03	20830066	T	G	nonsynonymous SNV	BnaA03G0387300ZS:BnaA03T0387300ZS:exon3:c.A789C:p.R263S,BnaA03G0387300ZS:BnaA03T0387300ZS.1:exon3:c.A771C:p.R257S,BnaA03G0387300ZS:BnaA03T0387300ZS.2:exon3:c.A789C:p.R263S,
BnaA03G0387300ZS	A03	20830089	T	C	nonsynonymous SNV	BnaA03G0387300ZS:BnaA03T0387300ZS:exon3:c.A766G:p.I256V,BnaA03G0387300ZS:BnaA03T0387300ZS.1:exon3:c.A748G:p.I250V,BnaA03G0387300ZS:BnaA03T0387300ZS.2:exon3:c.A766G:p.I256V,
BnaA03G0387300ZS	A03	20830095	C	T	nonsynonymous SNV	BnaA03G0387300ZS:BnaA03T0387300ZS:exon3:c.G760A:p.V254I,BnaA03G0387300ZS:BnaA03T0387300ZS.1:exon3:c.G742A:p.V248I,BnaA03G0387300ZS:BnaA03T0387300ZS.2:exon3:c.G760A:p.V254I,
BnaA03G0387300ZS	A03	20830364	A	G	nonsynonymous SNV	BnaA03G0387300ZS:BnaA03T0387300ZS:exon3:c.T491C:p.I164T,BnaA03G0387300ZS:BnaA03T0387300ZS.1:exon3:c.T473C:p.I158T,BnaA03G0387300ZS:BnaA03T0387300ZS.2:exon3:c.T491C:p.I164T,
BnaA03G0387900ZS	A03	20867684	G	A	nonsynonymous SNV	BnaA03G0387900ZS:BnaA03T0387900ZS:exon2:c.G118A:p.A40T,

Table S3. Cont.

ID	Chr	Pos	Ref	Alt	Annotation-effect	Annotation-effect detail
BnaA03G0388100ZS	A03	20877401	A	AAGCAG C	nonframeshift insertion	BnaA03G0388100ZS:BnaA03T0388100ZS:exon1:c.12_13insGCTGCT:p.S5delinsAAS,
BnaA03G0388100ZS	A03	20877388	T	C	nonsynonymous SNV	BnaA03G0388100ZS:BnaA03T0388100ZS:exon1:c.A26G:p.N9S,
BnaA03G0388500ZS	A03	20893985	C	T	nonsynonymous SNV	BnaA03G0388500ZS:BnaA03T0388500ZS:exon13:c.C1247T:p.T416M,
BnaA03G0388600ZS	A03	20898938	A	G	nonsynonymous SNV	BnaA03G0388600ZS:BnaA03T0388600ZS.1:exon7:c.A961G:p.T321A,BnaA03G0388600ZS:BnaA03T0388600ZS.2:exon7:c.A961G:p.T321A,BnaA03G0388600ZS:BnaA03T0388600ZS:exon7:c.A961G:p.T321A,
BnaA03G0388700ZS	A03	20902867	CTGCAT	C	splicing	BnaA03G0388700ZS
BnaA03G0388700ZS	A03	20904258	T	TA	splicing	BnaA03G0388700ZS(BnaA03T0388700ZS:exon5:c.1706-2->T)
BnaA03G0388800ZS	A03	20911240	A	C	nonsynonymous SNV	BnaA03G0388800ZS:BnaA03T0388800ZS:exon7:c.T1015G:p.S339A,
BnaA03G0388800ZS	A03	20913589	C	G	nonsynonymous SNV	BnaA03G0388800ZS:BnaA03T0388800ZS:exon1:c.G81C:p.E27D,
BnaA03G0388900ZS	A03	20916073	A	G	nonsynonymous SNV	BnaA03G0388900ZS:BnaA03T0388900ZS:exon2:c.A170G:p.N57S,
BnaA03G0388900ZS	A03	20916085	A	G	nonsynonymous SNV	BnaA03G0388900ZS:BnaA03T0388900ZS:exon2:c.A182G:p.N61S,
BnaA03G0388900ZS	A03	20916977	T	C	nonsynonymous SNV	BnaA03G0388900ZS:BnaA03T0388900ZS:exon4:c.T383C:p.M128T,
BnaA03G0388900ZS	A03	20917129	G	A	nonsynonymous SNV	BnaA03G0388900ZS:BnaA03T0388900ZS:exon4:c.G535A:p.G179S,

Table S3. Cont.

ID	Chr	Pos	Ref	Alt	Annotation-effect	Annotation-effect detail
BnaA03G0388900ZS	A03	20917274	G	A	nonsynonymous SNV	BnaA03G0388900ZS:BnaA03T0388900ZS:exon5:c.G598A:p.V200M,
BnaA03G0388900ZS	A03	20917275	T	G	nonsynonymous SNV	BnaA03G0388900ZS:BnaA03T0388900ZS:exon5:c.T599G:p.V200G,
BnaA03G0388900ZS	A03	20917291	G	A	nonsynonymous SNV	BnaA03G0388900ZS:BnaA03T0388900ZS:exon5:c.G615A:p.M205I,
BnaA03G0389100ZS	A03	20922184	G	C	nonsynonymous SNV	BnaA03G0389100ZS:BnaA03T0389100ZS:exon3:c.C343G:p.Q115E,
BnaA03G0389100ZS	A03	20922449	C	G	nonsynonymous SNV	BnaA03G0389100ZS:BnaA03T0389100ZS:exon2:c.G193C:p.E65Q,
BnaA03G0389100ZS	A03	20922472	T	C	nonsynonymous SNV	BnaA03G0389100ZS:BnaA03T0389100ZS:exon2:c.A170G:p.H57R,
BnaA03G0389300ZS	A03	20935312	G	T	nonsynonymous SNV	BnaA03G0389300ZS:BnaA03T0389300ZS:exon5:c.G618T:p.E206D,
BnaA03G0389300ZS	A03	20934774	GTA	G	splicing	BnaA03G0389300ZS
BnaA03G0389400ZS	A03	20944328	GGAA	G	nonframeshift deletion	BnaA03G0389400ZS:BnaA03T0389400ZS:exon24:c.3229_3231del:p.1077_1077del,
BnaA03G0389400ZS	A03	20940673	T	G	nonsynonymous SNV	BnaA03G0389400ZS:BnaA03T0389400ZS:exon11:c.T1179G:p.D393E,
BnaA03G0389400ZS	A03	20944269	A	C	nonsynonymous SNV	BnaA03G0389400ZS:BnaA03T0389400ZS:exon24:c.A3169C:p.K1057Q,
BnaA03G0389500ZS	A03	20946808	C	T	nonsynonymous SNV	BnaA03G0389500ZS:BnaA03T0389500ZS:exon3:c.C301T:p.L101F,
BnaA03G0389500ZS	A03	20947758	C	T	nonsynonymous SNV	BnaA03G0389500ZS:BnaA03T0389500ZS:exon5:c.C754T:p.L252F,
BnaA03G0389500ZS	A03	20947939	A	G	nonsynonymous SNV	BnaA03G0389500ZS:BnaA03T0389500ZS:exon5:c.A935G:p.N312S,

Table S3. Cont.

ID	Chr	Pos	Ref	Alt	Annotation-effect	Annotation-effect detail
BnaA03G0389600ZS	A03	20950886	C	T	nonsynonymous SNV	BnaA03G0389600ZS:BnaA03T0389600ZS:exon1:c.G245A:p.S82N,
BnaA03G0389600ZS	A03	20951081	G	C	nonsynonymous SNV	BnaA03G0389600ZS:BnaA03T0389600ZS:exon1:c.C50G:p.P17R,
BnaA03G0389600ZS	A03	20951095	C	G	nonsynonymous SNV	BnaA03G0389600ZS:BnaA03T0389600ZS:exon1:c.G36C:p.M12I,
BnaA03G0389600ZS	A03	20951120	G	A	nonsynonymous SNV	BnaA03G0389600ZS:BnaA03T0389600ZS:exon1:c.C11T:p.T4I,
