# Supplementary material for: Identification of Dwarfing Candidate Genes in Brassica napus L. LSW2018 through BSA–Seq and Genetic Mapping
Source: Plants (Basel). 2024 Aug 18;13(16):2298. doi: 10.3390/plants13162298 (PMC11359780; doi:10.3390/plants13162298)
Supplement: Supplementary file 1 [file plants-13-02298-s001.zip › Table S4 The relative expression of 42 genes in dwarf and high individuals.pdf]

Table S4. The relative expression of 42 genes in dwarf and high individuals

No.	Gene ID	Dwarf individuals Cq value	Mean expression（$2^{-\Delta CT}$）	SD	High individuals Cq value	Mean expression（$2^{-\Delta CT}$）	SD
1	BnaA03G0378200ZS	-	-	-	-	-	-
2	BnaA03G0378500ZS	24.34594381	0.297255966	0.060208086	23.37980803	1.290577112	0.241569899
3	BnaA03G0378800ZS	-	0.00011572	0.739650136	35.15833165	0.000367363	0.034890363
4	BnaA03G0379000ZS	29.38556145	0.009037629	0.201637827	28.0271626	0.051498056	0.141036404
5	BnaA03G0379100ZS	28.04797322	0.022840662	0.191002859	27.81248477	0.059760648	0.287255484
6	BnaA03G0379200ZS	-	-	-	-	-	-
7	BnaA03G0380500ZS	-	-	-	-	-	-
8	BnaA03G0381200ZS	30.50382711	0.004163159	0.163268413	30.34890026	0.010300971	0.154706094
9	BnaA03G0382800ZS	30.60276563	0.003887224	0.127321009	32.08580882	0.003090413	0.324418755
10	BnaA03G0384600ZS	23.26727023	0.62783234	0.538705776	31.81872103	0.00371893	0.242284604
11	BnaA03G0384700ZS	27.0457775	0.045750901	0.048313527	27.44274164	0.077218093	0.074150495
12	BnaA03G0384800ZS	-	-	-	-	-	-
13	BnaA03G0385100ZS	27.44655823	0.034653941	0.204221502	29.66252558	0.016576679	0.383662987
14	BnaA03G0385300ZS	26.95686864	0.04865908	0.204762113	27.96702343	0.053690139	0.335089519
15	BnaA03G0385400ZS	32.68359148	0.001393104	0.8097536	30.64216092	0.004320377	0.283696307
16	BnaA03G0385600ZS	29.55176435	0.012211174	0.102644422	27.62181854	0.035053818	0.12597149
17	BnaA03G0385700ZS	-	-	-	-	-	-
18	BnaA03G0385800ZS	31.0313885	0.00437871	0.023764912	30.4758683	0.004848202	0.382245206
19	BnaA03G0385900ZS	31.22928892	0.00381744	0.143071865	27.98577599	0.027237875	0.238828289
20	BnaA03G0386100ZS	28.90432802	0.019127367	0.084491109	28.25434217	0.022611363	0.256550632
21	BnaA03G0386200ZS	28.66803583	0.022531298	0.084538122	27.06061261	0.051721981	0.027152016
22	BnaA03G0386300ZS	29.77673853	0.010448007	1.914530833	28.82207638	0.015255334	0.27053465
23	BnaA03G0386400ZS	33.85247315	0.000619605	0.230851924	33.23420739	0.000716536	0.281797683
24	BnaA03G0386600ZS	-	-	-	-	-	-
25	BnaA03G0386800ZS	27.37272588	0.055298502	0.071039959	27.14282164	0.048857109	0.071171352
26	BnaA03G0386900ZS	29.33031017	0.014237108	0.064036026	29.09552688	0.012621324	0.267343985

27	BnaA03G0387000ZS	29.59131252	0.011880979	0.243794389	28.5712129	0.018152613	0.055182938

Table S4. Cont.

No.	Gene ID	Dwarf individuals Cq value	Mean expression（$2^{-\Delta CT}$）	SD	High individuals Cq value	Mean expression（$2^{-\Delta CT}$）	SD
28	BnaA03G0387100ZS	-	-	-	-	-	-
29	BnaA03G0387200ZS	21.65896857	1.558241986	0.170368484	19.59490099	8.704494927	0.149021961
30	BnaA03G0387300ZS	31.6357489	0.00154641	0.217799954	30.04513744	0.006221698	0.15266811
31	BnaA03G0387900ZS	-	-	-	-	-	-
32	BnaA03G0388100ZS	23.56215225	0.416600249	0.494331177	22.10787541	1.524975606	0.167242986
33	BnaA03G0388500ZS	31.30838982	0.001940303	0.687149752	30.39927573	0.004867463	0.294033018
34	BnaA03G0388600ZS	31.46242718	0.001743813	0.160732366	30.11349509	0.005933777	0.041128282
35	BnaA03G0388700ZS	-	-	-	-	-	-
36	BnaA03G0388800ZS	27.85076392	0.021316641	1.041570318	26.41916347	0.076813202	0.277897134
37	BnaA03G0388900ZS	-	-	-	-	-	-
38	BnaA03G0389100ZS	27.94409419	0.019981292	0.378121025	28.20617219	0.022258333	0.044462396
39	BnaA03G0389300ZS	25.10679208	0.142802761	0.80755825	24.75326759	0.243736461	0.416949718
40	BnaA03G0389400ZS	29.3328126	0.007630933	0.704644777	28.27826574	0.021173386	0.422237198
41	BnaA03G0389500ZS	30.83680981	0.002690478	0.79381719	29.11819554	0.011828952	0.025893216
42	BnaA03G0389600ZS	28.11078562	0.017801002	0.825143839	25.46889979	0.148420449	0.023999838
