# Supplementary material for: Identification of Dwarfing Candidate Genes in Brassica napus L. LSW2018 through BSA–Seq and Genetic Mapping
Source: Plants (Basel). 2024 Aug 18;13(16):2298. doi: 10.3390/plants13162298 (PMC11359780; doi:10.3390/plants13162298)
Supplement: Supplementary file 1 [file plants-13-02298-s001.zip › Table S5 The qRT -PCR primer of 42 genes.pdf]

Table S5. The qRT -PCR primer of 42 genes

Primer Name	Forward Primer Sequence (5′-3′)	Reverse Primer Sequence (5′-3′)
BnaA03G0378200ZS	CAGACTCAGACACTGCCTCTT	CACCACCGACAATGAAGTTCTT
BnaA03G0378500ZS	CTTGAGATGGCGAGGAAGGT	GAGGCAGAGGAATAGTGACAGA
BnaA03G0378800ZS	TCTGGTTCGGTTGGTATGGTT	GAGAGTGGTTGTAACTGCTGTC
BnaA03G0379000ZS	CACTCGGACGCTGGAGATTA	CACTCCTCATATCAGGCTCAGA
BnaA03G0379100ZS	CCTTGGTGGTGGAACAGAGT	GACGTGAACGCCTGAGACT
BnaA03G0379200ZS	ATCGAGCTGTGGATGATGAGA	TTCTTAACGGTAGCGGTGTCT
BnaA03G0380500ZS	TGGTGTCATCTATGGCAAGGA	CCAACGCAGACCAACGATAG
BnaA03G0381200ZS	AGGCAAGATTCACTCGTAACC	TCACTTTCTCGTCAACATTAGC
BnaA03G0382800ZS	GAACGAGAACGCTTGACCAA	CAGTCGCTAATGTTCCTCCAG
BnaA03G0384600ZS	CAGCAGGTTACTTCAACAATCA	TGCTTCACCGTTCTCACATAG
BnaA03G0384700ZS	TCCGTCGCAGTTCACAATC	ACATCAGAAGAAGAAGCAACAC
BnaA03G0384800ZS	TTGCCTCCTTGTGTCTATCAGT	CCGATCTCCACGCACCATAT
BnaA03G0385100ZS	CGAAGACACGGAAGAACTAAGG	ACACGCTGTTCTCCAAGTAGT
BnaA03G0385300ZS	CGGTGAGGAACAAGTTGAAGAA	CAGCAGTCGTATCTCCAGTGA
BnaA03G0385400ZS	CCTCTTCTTCTTCTTCCTCACA	TCATCAGGTGTCAGTCCTCTAA
BnaA03G0385600ZS	ACCAACAATTCCAGCATACTTG	AACCGACAGCACATCAGAAG
BnaA03G0385700ZS	CGGCTTCTTCAACGGTTCAA	CCTCATTAGACGCCTGTTCAAT
BnaA03G0385800ZS	GGTTGCCGCCACAATCAA	GGTCTGCCTTGAACGAAGAAT
BnaA03G0385900ZS	GCTCCTGCTCCTGTGGTTAG	CGTCTGTGCTGAGTTCTTGTG
BnaA03G0386100ZS	AGAGATGGACCGTATCATTAGC	TCTTGTAAGGCTCGTTGATGG
BnaA03G0386200ZS	CTGGCGAACCTCATGTAGAAG	TCATCATCAACACAGACTCCTT
BnaA03G0386300ZS	GTTACTCTCCGACCATTCATCC	CTCTTGACCCGCTGTTTCTTT
BnaA03G0386400ZS	GCTGGTTGTGAATGGACTTAT	TACTTGCTCTTCCTGACTGTA
BnaA03G0386600ZS	TATCCTCTTACGCCTTGATCCA	TACTTGCTCTTCCTGACTGTA
BnaA03G0386800ZS	GTGGTGCTGCTGTTCCTATC	TCGGTGGTTGATGTGTCTGA
BnaA03G0386900ZS	GTTGTTCTCTGGCTCTGCTTAA	TCTCACTTCTGCTCTGATTCCT
BnaA03G0387000ZS	TGTTGCTTCCATAACTGATGCT	TAGATGCCATCGTCGGTGAA
BnaA03G0387100ZS	CATGGTTAGACACGCACAGT	GAGCAGAAGTATTGGACCTGAG
BnaA03G0387200ZS	CGGAGACATTCGCTAAGAACC	CCTTGAACCACACGGCTTC
BnaA03G0387300ZS	AATGCCATGAGGAGTTGTGTTC	GCGGCTCTGGTTAATCAAGTT
BnaA03G0387900ZS	CCGTGGTGGTGGATATGGT	CCGTAGTAGCCTCTGTAGCA
BnaA03G0388100ZS	GGCGAGGATTACCACACCAT	ACGACTAGAGGAGCGAACAAG
BnaA03G0388500ZS	TGGAATGGCACCAGTTGTTG	CAAGCAGTAGCATCGGTAGTC
BnaA03G0388600ZS	GGTGAAGGCTAAGGCGACTT	ATCACAGGAGCGAGGAACAATA
BnaA03G0388700ZS	ATGGCTCTCATTACCGTCCTT	ACTGCTATCAACTCCTCTGGTT
BnaA03G0388800ZS	CGCCGCTATCTCCATAATCAC	GCTCCATCTCCATCGTTAGGT
BnaA03G0388900ZS	GGTTCTTCATATTCGGCATTCTC	TGTGTTCCTTCTCTACTCTCCAT
BnaA03G0389100ZS	TATGGAGGAAGAGGTGGTTATGG	GTGTTGAGGAGGAGGCTTGA
BnaA03G0389300ZS	TCTCAACTCCAAGGTGTATCTC	GGTTCATCTGCCTGTTCATCT
BnaA03G0389400ZS	AAGCCTCCAGGATCAATATGTGT	CGTCCTCAGAAGTGCCTAACC

Table S5. Cont.

Primer Name	Forward Primer Sequence (5′-3′)	Reverse Primer Sequence (5′-3′)
BnaA03G0389500ZS	TACGCTCAACGCACCACTC	TGCCTCTACTTGTTCCTCTGAAT
BnaA03G0389600ZS	GCAAGGCTTCTTCTGGACTTC	ACCAGCAGCAGCAAGAGATT
BnaA03G0378200ZS	CAGACTCAGACACTGCCTCTT	CACCACCGACAATGAAGTTCTT
BnaA03G0378500ZS	CTTGAGATGGCGAGGAAGGT	GAGGCAGAGGAATAGTGACAGA
BnaA03G0378800ZS	TCTGGTTCGGTTGGTATGGTT	GAGAGTGGTTGTAACTGCTGTC
BnACTIN7	CGCGCCTAGCAGCATGAA	GTTGGAAAGTGCTGAGAGATGCA
